# Resolving the Ionotropic P2X4 Receptor Mystery Points towards a New Therapeutic Target for Cardiovascular Diseases

**DOI:** 10.3390/ijms21145005

**Published:** 2020-07-15

**Authors:** Bruno Bragança, Paulo Correia-de-Sá

**Affiliations:** 1 Laboratório de Farmacologia e Neurobiologia, Center for Drug Discovery and Innovative Medicines (MedInUP), Instituto de Ciências Biomédicas de Abel Salazar, Universidade do Porto, R. Jorge Viterbo Ferreira, 228, 4050-313 Porto, Portugal; bbraganca14@hotmail.fr; 2 Department of Cardiology, Centro Hospitalar Tâmega e Sousa, 4564-007 Penafiel, Portugal

**Keywords:** ATP, cardiovascular system, P2X4 receptor

## Abstract

Adenosine triphosphate (ATP) is a primordial versatile autacoid that changes its role from an intracellular energy saver to a signaling molecule once released to the extracellular milieu. Extracellular ATP and its adenosine metabolite are the main activators of the P2 and P1 purinoceptor families, respectively. Mounting evidence suggests that the ionotropic P2X4 receptor (P2X4R) plays pivotal roles in the regulation of the cardiovascular system, yet further therapeutic advances have been hampered by the lack of selective P2X4R agonists. In this review, we provide the state of the art of the P2X4R activity in the cardiovascular system. We also discuss the role of P2X4R activation in kidney and lungs vis a vis their interplay to control cardiovascular functions and dysfunctions, including putative adverse effects emerging from P2X4R activation. Gathering this information may prompt further development of selective P2X4R agonists and its translation to the clinical practice.

## 1. The “Purinome”

The concept of “purinome” encompasses the molecular machinery necessary for purinergic signaling. The core of this system is adenine nucleotides and nucleosides and their activity over a wide range of transmembrane purinoceptors. Terms like “purinergic nerves” and “purinoceptors” were coined in the early 1970s after Geoffrey Burnstock, one of the most widely cited scientists who recently (2 June 2020) passed away at the age of 91. The purinoceptor family is further subdivided into P1 and P2 receptor subtypes according to their sensitivity to adenosine and adenine and uracil nucleotides (e.g., ATP, ADP, UTP, UDP, UDP-glucose), respectively. Four subtypes of adenosine-sensitive G protein-coupled P1 receptors have been cloned, A_1_, A_2A_, A_2B_, and A_3_. Regarding the P2 receptor family, it is sub-classified into seven ionotropic P2X receptors (P2X1-7) and eight G-protein-coupled metabotropic P2Y receptors (P2Y_1,2,4,6,11,12,13,14_). Each purinoceptor has a specific pharmacological profile and biological fingerprint, which allows its identification overtaking the scarcity of selective pharmacological tools [1]. Besides lytic mechanisms that occur after cellular damage, the release of ATP and other purines/pyrimidines may be elicited by multiple stimuli (e.g., hypoxia, inflammation, mechanical stimuli) and occur via highly regulated processes, such as vesicle exocytosis and diffusion through plasma membrane channels [2,3]. Once in the extracellular milieu, ATP is sequentially hydrolyzed to adenosine by a cascade of plasma membrane-bound nucleotidases (e.g., NTPDase1/CD39, ecto-5′-nucleotidase/CD73), shifting cellular signaling from P2- to P1-mediated responses. Then, adenosine follows one of two removal pathways: (1) cellular reuptake by nucleoside transporters or (2) enzymatic deamination to inosine by adenosine deaminase [4].

The relevance of purinergic signaling to control cardiovascular functions is indisputable, spanning from the pioneering work of Drury and Szent-Györgyi in 1929 on the role of adenosine in the mammalian heart [5] to more recent reports leading to the use of P2Y_12_ receptor antagonists to reduce platelet aggregation in thrombotic diseases (e.g., myocardial infarction, stroke) [6,7]. Notwithstanding this, drugs other than adenosine (and its derivatives) and P2Y_12_ receptor antagonists have already entered clinical trials for cardiovascular diseases, with some other drugs still under development and awaiting clearance to enter the clinical trial pipeline [8].

Nowadays, there is an increasing enthusiasm for the modulation of P2X purinoceptors functions in health and disease, with the P2X7R as the most promising candidate confirmed by successfully completed clinical trials. More recently, the P2X4R has emerged as another promising drug target. Despite the lack of selective P2X4R agonists available so far, modulation of the P2X4R has been demonstrated to be beneficial in cardiac diseases, pain sensation, cancer, and inflammatory diseases [8,9]. Due to these putative therapeutic hints, we expect a boost in the development of selective P2X4R agonists in the forthcoming years. This prompted us to review the recent advances in deciphering the pathophysiological relevance of the P2X4R in the cardiovascular system. Considering that impairment of other body systems (e.g., renal and respiratory) might have a huge impact in the management and prognosis of cardiac patients, we thought it was useful to discuss the (beneficial and/or detrimental) repercussions of the P2X4R activation in these territories.

## 2. The P2X4 Receptor: From Molecular Characteristics to Its Modes of Action

The P2X4R was the first crystal structure of a P2X receptor to be resolved [10]. Sequence homology of P2X4R in mammals exceeds 85%, with some genetic variants been described for this receptor [11,12]. The P2X4R results from the assembly of three subunits organized in a dolphin-like structure. Each subunit has two membrane-spanning domains, with C- and N-terminals located intracellularly, and a large ectodomain for ATP binding [10]. Structural homology among P2X receptors allows different assemblies of P2X subunits. Heterotrimeric P2X receptors have different pharmacological and functional properties from homotrimeric versions [13]. Heterotrimers have been described for P2X4R, particularly in combination with P2X6R subunits; however, it remains to be clarified whether they exist in native systems [13,14].

P2X4R orthosteric binding sites can be found between two adjacent subunits, which means that each P2X4R can harbor up to three ATP molecules [9,15]; the pEC_50_ for ATP of the Human P2X4R is in the low micromolar range (Table 1) [16]. The P2X4R ATP-binding site lacks some specificity for the purine base allowing binding of other nucleotides including non-adenine compounds, such as cytidine triphosphate (CTP); the P2X4R exhibits low affinity (millimolar range) for this pyrimidine [17]. Similarly to other P2X receptors, the P2X4R has two main conformational states: closed and open. Occupancy of the orthosteric binding pocket by ATP and other agonists induces structural changes that open a permeation pore for Na^+^, K^+^, and Ca^2+^ ions [15,18]. Opening of P2X4R generates a depolarizing inward current, with a fourfold permeability for Ca^2+^ in relation to Na^+^ (P_Ca_/P_Na_ ≈ 4), contributing to the activation of several Ca^2+^-dependent intracellular signaling processes [18,19]. The P2X4R reversion potential is around 0 millivolts, which means that P2X4R-mediated currents easily become outward at positive potentials [20,21]. Interestingly, two P2X4R permeant conformations seem to exist regarding the dimensions of the channel pore (small vs. large) [19,20,22]. Indeed, the P2X4R may share some permeant characteristics with the P2X7R, including the formation of a high-permeability pore that allows passage of large molecules, including large fluorescent dyes. This high-permeant state of the P2X4R exists on its own, without needing the accessory proteins (e.g., pannexin 1) required to form the high-permeability pore together with the P2X7R [22,23]. Like its P2X7R sibling, the P2X4R large pore formation is favored by low extracellular Ca^2+^ and is regulated by phosphatidylinositol 4,5-bisphosphate (PIP2) content in the plasma membrane [23,24].

The role of the P2X receptor in several physiological processes has been hindered by the current lack of selective agonists and antagonists. This limitation has been partially overridden by the pharmacological characterization of agonist profiles, gating kinetics, and function in comparison with other P2X receptors [20]. The P2X4R is a fast purinergic excitatory receptor, and in comparison with other P2X receptors, it exhibits intermediate sensitivity for ATP (≈10µM) and desensitization time course in rodents [20,22,65]. Synthetic ATP derivatives (e.g., αβ-methylene ATP (αβ-meATP); βγ-methyleneATP (βγ-meATP); adenosine 5′-O-(3-thio)triphosphate (ATPγS), 2-MethylthioATP (2-meSATP), 2,3-O-(4-benzoyl-benzoyl)ATP (BzATP)) activate all P2X receptors, yet, contrary to other P2X receptor subtypes, the P2X4R is relatively insensitive to αβ-meATP in rodents (Table 1). The agonist rank potency order for the P2X4R is as follows: ATP > ATPγS > 2-meSATP ≥ CTP > αβ-meATP [20,66]. The list of available P2X4R antagonists is gradually expanding with some compounds presenting well-acceptable selectivity over other P2X receptor subtypes, like 5-BDBD (5-(3-bromophenyl)-1,3-dihydro-(1)benzofuro(3,2-e)(1,4)diazepin-2-one; pIC50~5-6) [11,20,64]. Relative insensitivity to pyridoxalphosphate-6-azophenyl-2′,4′-disulfonic acid (PPADS), and suramin, two broad-spectrum P2 antagonists, is another pharmacological characteristic of the P2X4R (Table 1) [20].

Regulation of the P2X4R activity is complex and not limited to binding and unbinding of ATP [9,20,67]. Trace metals are important modulators of the P2X4R activity, and their role may be used as a strategy for this receptor characterization (Table 1). For instance, zinc ions (Zn^2+^) bind to the “M1 site” of the P2X4R and increase its affinity for ATP [66,68]. Conversely, copper ions (Cu^2+^) inhibit the P2X4R maximal response without affecting its affinity for ATP [20]. This receptor is also sensitive to pH, a property derived from the surface lining of protonable histidines. In acidic conditions, like those occurring during ischemia or inflammation, protons (H^+^) inhibit the P2X4R activity [67,69]. Allosteric modulators are other important pharmacological tools. This is headed by the prototypical positive allosteric modulator of the P2X4R, ivermectin, which highly increases the ATP agonist potency [67,70]. Other allosteric modulators have been described, which include several commonly used drugs like paroxetine, alcohol, and propofol [20,41,57,64]. The C-terminal of the P2X4R is a target for kinase regulation, namely by protein kinase A (PKA), which increases the receptor activity. Rather than a direct regulation by phosphorylation, PKA stimulation recruits accessory proteins to bind to the C-terminal of the P2X4R [71]. The P2X4R is also sensitive to the composition of plasma membrane; for instance, depletion of membrane PIP2 by stimulation of phospholipase C (PLC) decreases the current induced by the receptor and slows its recovery from desensitization [72].

Gene expression and cellular localization represent another level of regulation of the P2X4R. This receptor is present in multiple cells throughout the body, but its relative expression depends on cell types, development status, and health/disease conditions [20,21,66]. Expression of the P2X4R is highly sensitive to certain stimuli, including hypoxia [73,74] and inflammation [75], and is controlled by specific transcription factors. STAT1, GATA-2, and IRF-5 transcription factors are known activators of the P2X4R gene promoter [49,75,76]. Trafficking of the P2X4R to the plasma membrane is paramount to sense extracellular ATP, while internalization represents an important negative feedback mechanism to avoid overactivation. The C-terminal tail of the P2X4R is a key element in desensitization and internalization processes; it functions as an endocytic-sorting signal to lysosomes [77]. Intracellular localization of the P2X4R has increasingly been demonstrated in certain cell types; localization of this receptor in vesicles and lysosomes supports its role in vesicle turnover, as demonstrated concerning exocytosis [11,78,79].

## 3. Benefits from the P2X4 Receptor Activation in the Heart

Cardiac output is a function of heart rate and stroke volume, with the latter being influenced by pre-load, after-load, and inotropy. ATP and other purines regulate all these features. For decades it was generally accepted that intravenous infusion of ATP decreased cardiac works by means of its breakdown to adenosine via activation of the inhibitory A_1_ receptor. With experimental advances, the “true” ATP effects soon became resolved, and now there is plenty of evidence to support involvement of P2 receptors in acute and chronic physiological processes in the heart. P2 receptors are expressed not only in the working myocardium, but also in other cardiac cells [80,81,82]. Among P2X receptors, the P2X4R is the most expressed receptor subtype in the heart, particularly in supraventricular tissues [83,84]. The P2X4R is distributed in various cardiac cells, including cardiomyocytes, endothelial and smooth muscle cells of cardiac blood vessels, cells of the cardiac conduction system [83,85], intracardiac neurons [86], and cardiac fibroblasts [87].

ATP, through activation of P2 purinoceptors, increases cardiac inotropy. Although some conflicting results exist regarding the receptor involved in this effect, accumulating evidence suggests an involvement of P2X receptors, particularly the P2X4R subtype [81]. In 2001, a seminal study from B.T. Liang’s group demonstrated that cardiac preparations overexpressing the human P2X4R exhibited increased contractile performance when challenged with the P2X receptor agonist, 2-meSATP. Remarkably, baseline cardiac output was also incremented, but no effects were observed regarding chronotropy and/or ventricular remodeling [88]. Later on, the same group showed that P2X4R overexpression was indeed protective by increasing survival both in ischemic and in non-ischemic models of heart failure. This theory was strengthened by finding that overexpression of the P2X4R favors rescuing of several hallmarks of heart failure (HF), namely ventricular systolic dysfunction, β-adrenergic desensitization, hypertrophy, and maladaptive remodeling [89,90,91]. Interestingly, HF and coronary artery disease are on their own associated with upregulation of the P2X4R expression in the heart [33,83]. In a rodent model of hypoxia-induced pulmonary hypertension, the P2X4R was found to be upregulated in the right ventricle [73]. Likewise, a compensatory upregulation of P2X currents was observed in the heart of animals exhibiting cardiomyopathy due to calsequestrin overexpression; in this animal model stimulation of P2X receptors with MRS-2339, a charged methanocarba derivative of 2-Cl-AMP resulted in reversion of maladaptive cardiac remodeling and prolonged survival [33]. The recently developed diester-masked uncharged phosphonate, MRS-2978, a compound structurally related with MRS-2339 but chemically modified to be orally bioavailable and more resistant to enzymatic hydrolysis, presents benefits in improving cardiac dysfunction in both ischemic and pressure-overload HF animal models [34,53]. Conversely, knocking down the P2X4R is normally associated with deterioration of cardiac ventricular function and progression to HF [92]. Data, thus, indicate that stimulation of the P2X4R ameliorates cardiac contractile performance by increasing inotropy and by favoring the recruitment of protective signaling mechanisms.

**Figure 1:**
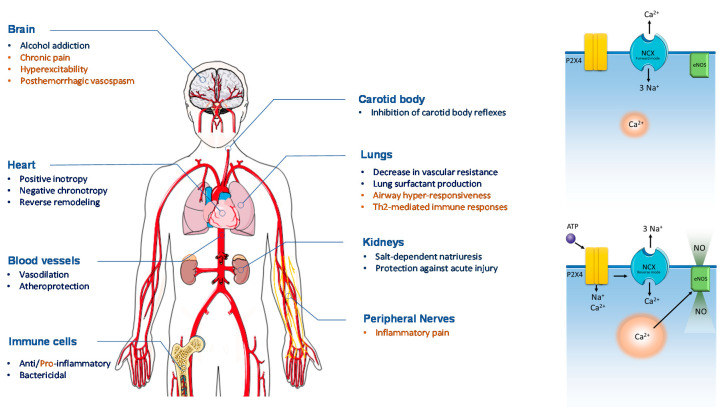
**Schematic overview of systemic P2X4R stimulation.** An overall cardioprotective effect is observed with P2X4R stimulation. Permeation of Na^+^ and Ca^2+^ ions through P2X4R interferes with the forward mode of the Na^+^-Ca^2+^ exchanger (NCX) and Ca^2+^-dependent activation of endothelial nitric oxide synthase (eNOS) (right panels). In the heart, stimulation of P2X4R improves cardiac muscle contraction and optimizes myocardial energy supply vs. demand by reducing chronotropy. The diuretic effect of P2X4R activation together with its vasodilation properties in both systemic and pulmonary arteries optimizes preload and afterload and, thereby, cardiac output in HF. Chronic activation of the P2X4R reverses maladaptive cardiac remodeling and improves the outcome of HF. Future P2X4R agonist therapies must deal with the wide P2X4R distribution throughout the human body resulting in putative unwanted side-effects (marked in orange). Figure composition used elements from Servier Medical Art (https://smart.servier.com).

As aforementioned, the P2X4R activation increases Ca^2+^ influx into the cells [18,19,66]. Ca^2+^ transients induced by P2X4R activation further stimulate massive Ca^2+^ release from sarcoplasmic reticulum through a mechanism of calcium-induced calcium release, which might explain the positive inotropic effect of this receptor [93]. It has also been demonstrated that the P2X4R modulates the activity of the Na^+^-Ca^2+^ exchanger (NCX), which contributes to prolonging intracellular Ca^2+^ accumulation and signaling. In ventricular myocytes from both wild-type and P2X4R-overexpressing mice, 2-meSATP caused an inward Na^+^ current which was high enough (about 1 mM) to potentiate Ca^2+^-entry via the NCX functioning in the reverse mode [94]. These findings led to the conclusion that Na^+^ influx via the P2X4R may putatively interfere with normal function of NCX, thus favoring intracellular Ca^2+^ accumulation (Figure 1).

Recently, our group demonstrated that in the sinoatrial node (SAN), the first element of the cardiac conduction system, the P2X4R interferes with the Ca^2+^-extrusion (forward) mode of the NCX [85], which constitutes a fundamental pacemaking mechanism to drive spontaneous SAN depolarization and to set sinus rhythm [95]. Our study demonstrated for the first time that rather than requiring its breakdown to adenosine, ATP on its own is able to decrease chronotropy via P2X4R activation. The dual role of the P2X4R in decreasing chronotropy while increasing inotropy strikingly resembles the effect of Na^+^/K^+^-ATPase inhibition by cardiac glycosides (e.g., digoxin) [85,96], expanding the clinical usefulness of P2X4R agonists to the treatment of tachyarrhythmias and congestive HF [97,98,99]. In support of this mechanism, istaroxime, a newer intracellular Na^+^ enhancer, has been demonstrated to improve both diastolic and systolic function in patients with decompensated HF with reduced ejection fraction (HFrEF), along with increasing systolic blood pressure while reducing heart rate, with no evidence of increases in the probability of cardiac arrhythmias [100,101,102,103]. Notwithstanding this, a note of caution must be taken concerning intracellular Na^+^ overload as a consequence of chronic P2X4R activation, since increases in diastolic Ca^2+^ concentration may worsen the diastolic dysfunction in HF conditions [104]. Another caveat is related to rhythm disturbances that may arise from perturbations of Na^+^ and Ca^2+^-handling resulting from prolonged activation of the P2X4R. As a matter of fact, some reports ascribed a pro-arrhythmic potential to ATP [105,106], which deserves to be further explored in future studies.

Apart from its key role in cardiac contraction and heartbeat initiation, Ca^2+^ is an important intracellular second messenger [107]. Yang and coworkers demonstrated that cardiac protection triggered by the P2X4R involves stimulation of endothelial synthesis of nitric oxide (NO) [92]. Synthesis of NO is a downstream target of Ca^2+^ signaling. The beneficial effects of NO in the cardiovascular system are well-known, which include vasodilation, inhibition of platelet aggregation, and regulation Ca^2+^ homeostasis [108]. Putative reversion of pathological cardiac remodeling may also be owed to NO production (Figure 1), though the P2X4R activation has been linked to proliferation and migration of cultured human cardiac fibroblasts, but this was only reported once several years ago [87]. Clearly, further studies are also needed to clarify the role of P2X4R in cardiac remodeling.

## 4. Cardiovascular Risk Factors: Is the P2X4 Receptor Friend or Foe?

Hypertension, diabetes, obesity, and dyslipidemia have a close relationship with heart diseases, major cardiovascular events, and death [109]. Strict control of these modifiable cardiovascular risk factors has a major impact on patients’ prognosis [30,110,111]. Not controlling these risk factors engage the cardiovascular disease continuum driven by atherosclerosis and inflammation [112].

Purinergic receptors have been targeted for the treatment of diabetes, hypertension, and other conditions associated with the metabolic syndrome [113,114,115]. Purines regulate multiple functions in blood vessels, ranging from short-lived mechanisms to more sustained processes, including those involved in maladaptive remodeling of hypertension and atherosclerosis [81]. The net vascular response depends on where and when the purinergic transmission occurs. Like acetylcholine (ACh), ATP has a dual response in controlling blood vessel tone, with both vasodilatory or vasoconstrictor responses [81,116]. One important source of ATP is provided from sympathetic nerves surrounding blood vessels. Stimulation of sympathetic nerves releases norepinephrine and ATP in the smooth muscle layer of blood vessels; these two potent vasoconstrictors are upregulated in hypertension [117]. ATP exerts transient vasoconstriction mostly through activation of the P2X1R on vascular smooth muscle cells (VSMCs) [118,119], followed by a smaller yet sustained contraction via P2X4R activation [120]. Both P2X1R and P2X4R localized on VSMCs contribute to vasospasm occurring as a consequence of hemorrhagic stroke [120].

When acting at the luminal side of intact blood vessels, circulating ATP causes vasodilation through activation of endothelial metabotropic P2Y_1_, P2Y_2_, P2Y_4_, and P2Y_6_ receptor subtypes; while the P2Y_1_ receptor is more sensitive to the ATP metabolite, ADP, the P2Y_2_, P2Y_4_, and P2Y_6_ receptor subtypes are mainly activated by uracil nucleotides, namely UTP and UDP, respectively. Increasing evidence shows that endothelial ATP-sensitive ionotropic P2X4R also participates in vasodilation. These receptors are particularly abundant in coronary and cerebral arteries [119]. Noteworthy is that endothelial cells release huge amounts of ATP in response to increased blood pressure/flow [121], thus creating a “self-regenerative ATP activation wave” spreading along endothelial cells in the vicinity [81]. In this sense, endothelial P2X4R may act as immediate mechano-transducers to re-establish local blood pressure/flow by promoting the endothelial release of vasodilation signaling mediators like NO [122]. This hypothesis is strengthened by findings linking hypertensive and vascular remodeling phenotypes to knockdown or loss-of-function mutations of the P2X4R [12,122]. For instance, the loss-of-function tyrosine-to-cysteine polymorphism in the 315 position of the human P2X4R ectodomain (Tyr 315 > Cys) was associated with higher blood pressure and reduced arterial compliance as a result of impaired endothelium-dependent vasodilation in large arteries [12]. Pathological alterations of renal P2X4R may also contribute to arterial hypertension (see below).

Apart from the local control, vascular tone can be also regulated centrally through cardiovascular reflexes involving carotid bodies (CBs) and aortic bodies (ABs). In CB, a cluster of chemoreceptor cells releases excitatory ATP in response to several chemostimulants such as hypoxia and hypercapnia, which then stimulates P2X2/3R located in sensory afferents of the carotid sinus nerve (CSN), a branch of the petrosal nerve. Firing of the CSN will then be integrated centrally at the brainstem to increase ventilation and to cause bradycardia and peripheral vasoconstriction [123]. Feedback inhibition of CB-mediated responses involves nitrergic nerves from the glossopharyngeal nerve (GPN). Interestingly, these inhibitory nitrergic nerves can be stimulated by several ATP-sensitive P2X receptors, namely P2X2/3R, P2X4R, and P2X7R, but contrary to the P2X2/3R that is present both in afferent and in efferent nerves, the P2X4R exclusively mediates the inhibitory pathway [123,124]. Hence, selective stimulation of the CB inhibitory drive with P2X4R agonists or blockage of P2X2/3R-mediated CB excitation may represent putative targets to control cardiorespiratory reflexes, particularly in patients who are refractory to common anti-hypertensive drugs [125,126]. Noteworthy, overactivation of CB is also observed in obstructive sleep apnea and type 2 diabetes and may represent a pivotal element in the vicious cycle causing drug-refractory hypertension and type 2 diabetes [126].

Atherosclerosis is a low-grade chronic inflammatory process that develops upon persistence of one or more of the aforementioned vascular risk factors [127]. ATP, acting mainly via metabotropic P2Y receptors, has potent mitogenic and pro-inflammatory effects involved in stepwise formation of atheroma plaques [81]. Evidence also exists on direct involvement of P2X receptors in atherosclerosis [81,128,129]. For instance, upregulation of endothelial P2X4R and/or P2X7R has been observed in response to cellular damage inflicted by dyslipidemia and hyperglycemia; overactivation of these receptors has been linked to the release of pro-inflammatory cytokines and increases in endothelial permeability [130]. Moreover, overexpression of the P2X4R in medial and neointima of rabbit aorta may ascribe the pro-atherosclerotic role of ATP in this vessel [131].

In primary monocyte-derived human macrophages, the P2X4R stimulates the release of CXCL5, a pro-inflammatory chemokine involved in neutrophil recruitment [132]. Nevertheless, caution must be taken in attributing a deleterious role to the P2X4R in atherosclerosis development, because increased production of endothelial CXCL5 was protective against macrophage foam cell accumulation in atherosclerotic plaques by a mechanism that involves cholesterol efflux from macrophages in a rodent model of atherosclerosis [133]. Moreover, the P2X4R was responsible for increases in Krüppel-like factor 2 (KLF2), an atheroprotective transcription factor [134]. 

As atherosclerotic plaques grow, the lumen of blood vessels becomes narrow, thus limiting blood flow and distal delivery of oxygen and nutrients. At this stage of atherosclerosis development, blood-flow-mediated shear stress dramatically increases along with overactivation of endothelial P2X4R (see above). In this context, activation of endothelial P2X4R engages protective mechanisms against ischemia in blood vessels where its density is higher, like coronary and cerebral arteries; this results in the increased expression of osteopontin, a well-known neuroprotective factor [135]. In the late stage of atherosclerosis, erosion and plaque rupture occurs leading to endothelial cells damage and inability to prevent thrombotic events, thus prompting acute coronary and stroke events. Endothelial damage exposes not only the procoagulant and platelet-aggregating features of subendothelial matrix proteins [6] but also the vasoconstrictive P2X1R and P2X4R on VSMCs to surplus amounts of ATP derived from platelet aggregation and damaged cells [119,120]. Thus, one may hypothesize that the P2X4R exerts a protective role in blood vessels when the endothelium is functional, but its action is detrimental once blood vessels are severely damaged, such as in acute coronary and aortic syndromes, as well as in stroke.

## 5. Role Played by the P2X4 Receptor in Renal Function and Dysfunction

Renal integrity and function are of utmost importance in patients with cardiovascular diseases. Acute and chronic kidney diseases have a negative impact on the prognosis of patients with HF and coronary artery diseases [136,137,138]. Multiple factors are involved in the cardiorenal axis regulation. These include neurohormonal mediators and the autonomic nervous system [139]. Renal purinoceptors control blood flow and glomerular filtration, which are main determinants of the water-electrolyte balance [140].

In healthy conditions, myogenic and tubule–glomerular feedback (TFG) mechanisms ensure that renal blood flow matches glomerular filtration rate (GFR) for proper water and solute handling. TFG is orchestrated by macula densa cells in distal tubules. These cells react to low NaCl levels in the lumen of distal tubules by favoring dilation of afferent arterioles and renin–angiotensin-dependent constriction of efferent arterioles (reviewed in [141]). Infusion of ATP into the renal artery exerts a dual effect in afferent arterioles that is dependent on the vascular tone. Upon increasing renal vascular resistance, ATP effects shift from P2X1R-mediated vasoconstriction to P2Y/P2X4R-induced NO-mediated vasodilation [140,142,143]. Blood filtration yields the urinary ultrafiltrate, and its composition changes as it moves throughout renal tubules. Multiple P2 receptors are expressed in renal tubules, and their activation promotes natriuresis through inhibition of several sodium transporters [144]. Interestingly, after surgical renal denervation for treatment of drug-refractory hypertension, the P2-mediated natriuresis is significantly favored [145]. Although the P2X4R is abundantly expressed in all renal segments, its functions are better characterized in distal segments of the nephron [140]. Activation of the P2X4R in this region contributes to modulate the activity of epithelial sodium channels (ENaC) [146]. However, regulation of ENaC activity by the P2X4R is not linear, and ATP may exert a dual role depending on sodium and body fluid composition. In low-salt conditions, stimulation of the P2X4R increases ENaC activity, while in salt-overload conditions the P2X4R exerts an opposite effect and promotes natriuresis [147,148]. As mentioned before, P2X4R^-^null mice exhibit a hypertensive phenotype [148]. Therefore, it is tempting to speculate that in patients with essential hypertension, P2X4R agonists may contribute to promote natriuresis and to lower blood pressure.

Acute kidney injury (AKI) inflicts cardiovascular damage by means of local inflammation, altered mitochondrial function, and the development of local fibrosis [138]. Inflammation-induced P2 X7R receptor activation display an important role in renal disease progression, with some properties being shared by its counterpart P2X4R [149]. In a rodent model of ischemic AKI, activation of the P2X4R exacerbates tubular necrosis and NOD-like receptor 3 (NLRP3)-dependent inflammation [150]. In contrast to the cardiac P2X4R, the activation of which putatively favors fibrosis [87], the renal P2X4R seems to have an antifibrotic role [151].

Chronic elevation of intraglomerular pressure caused by arterial hypertension is accompanied by proteinuria, as a consequence of podocyte damage. Proteinuria is a surrogate marker of cardiovascular diseases [152]. Podocytes are sensitive to intraglomerular pressure, and they are important elements of the glomerular filtration barrier. Interestingly, podocytes are endowed with P2X4Rs operating mechanotransduction by interacting with podocin and highly enriched cholesterol domains [153]]. Microalbuminuria is a hallmark of diabetic nephropathy, the progression of which has been associated with P2X4R activation and downstream stimulation of NLP3 inflammasome in podocytes [154]. However, it still remains to be clarified whether the mechanosensitive role of the P2X4R in podocytes has a protective or deleterious effect in the progression to glomerulosclerosis secondary to hypertension and diabetes.

## 6. Is the P2X4 Receptor Activation Beneficial or Detrimental in the Lung?

Heart and lungs operate as a coupled unit, with numerous hemodynamic and neurohormonal interactions between them. Pulmonary diseases increase morbidity and worsen survival in patients with cardiovascular diseases. Pulmonary arterial hypertension (PAH) is a prototypic disease model of pulmonary and cardiac functions’ interdependence. In PAH, there is a disproportionate increase in pulmonary vascular resistance (PVR), resulting in maladaptive remodeling of the right ventricle (RV) and HF [155]. Pulmonary vasodilators decrease PVR and improve PAH, particularly in group 1 PAH patients [156]. ATP and its metabolite, adenosine, have important roles in controlling pulmonary vascular tone. Similarly to that occurring in other vascular beds, ATP has both vasodilatory and vasoconstrictive effects in pulmonary arteries. Pulmonary vasoconstriction caused by ATP depends on activation of P2X1, P2Y_2,_ and P2Y_4_ receptors on VSMCs [157,158,159,160], while vasodilation prevails when it activates endothelial-located metabotropic P2Y_1_, P2Y_2,_ P2Y_4_, and/or ionotropic P2X4R [157,158,159,160]. As aforementioned, the pulmonary arteries express high levels of the P2X4R [161,162], where it seems to mediate shear-stress-induced ATP vasodilation [119,160]. Despite the fact that it has been demonstrated that intravenous delivery of P2X4R agonists has the potential to cause pulmonary vasodilation and to decrease RV overload, this warrants further investigations in disease models of PAH.

Respiratory infections and exacerbation of chronic lung diseases are common causes of acute decompensated heart failure (ADHF) [162,163]. Moreover, pulmonary congestion in ADHF often accompanies with ventilation/perfusion mismatch, which intensifies respiratory insufficiency with hypoxemia and hypercapnia, a situation that is already compromised in patients with underlying chronic pulmonary disease. As mentioned above, chronic hypoxemia, as well as respiratory acidosis, stimulate peripheral chemoreceptors and trigger several cardiovascular reflexes, accompanied by deleterious sympathetic overactivation and abnormal handling of water and natriuresis [123,155]. Most patients presenting with ADHF usually require diuretic, vasodilatory, and inotropic therapies to reduce congestion and optimize respiratory function and cardiac output [99]. Considering the proposed framework for the P2X4R in cardiovascular diseases, development of new and better-tolerated agonists for this receptor may represent an opportunity to improve ADHF patients’ symptomatology, as they would reduce pulmonary congestion subsequently to increases in cardiac inotropy, diuresis, and vasodilation.

Apart from endothelial-induced vasodilation of pulmonary vessels and decrease of right ventricle afterload, prolonged P2X4R activation has been associated with inflammation, hyperreactivity, and mucus production in the airways of susceptible individuals [164,165,166]. Chronic lung diseases usually have in their background an inflammatory response that precipitates deterioration of both cardiac and respiratory functions [167,168]. Extracellular ATP raises dramatically during inflammatory conditions to levels high enough to activate P2X7, P2Y_2_, and P2Y_6_ receptors, which normally exhibit low affinity for the nucleotide (see Section 2). Activation of these receptors is often associated with tissue fibrosis, polymorphonuclear leukocytes infiltration, and other immune cell activation, which are hallmarks of maladaptive pulmonary remodeling [167]. Indeed, termination of P2 purinoceptors-mediated signaling by enzymatic conversion of ATP to adenosine revealed to be protective in lung injury [169].

Although the P2X4R is present in immune cells from both myeloid and lymphoid lineages [166], and its expression is upregulated in inflammatory conditions [170], there is still no consensus about its role in inflammation given that both pro- and anti-inflammatory effects have been described in the literature [166,171,172,173,174]. In a murine experimental model of asthma, a positive correlation between P2X4R levels and inflammation severity has been found; blockage of P2X4R activation reversed some asthma remodeling hallmarks, including bronchoalveolar lavage fluid eosinophilia, peri-bronchial inflammation, Th2 cytokine production, and bronchial hyper-responsiveness [173,175]. Recently, it was demonstrated that stimulation of the P2X4R favors T cell migration to the lungs, whereas blockage of this receptor is protective against acute rejection of lung allotransplants [176]. The pro-inflammatory roles of the P2X4R are, in part, due to its expression in leucocytes, namely lymphocytes and eosinophils [176,177]. Moreover, it seems that ATP-induced mast cell degranulation involves the P2X4R activation [42], and this receptor is also implicated in the contraction of airway smooth muscle cells [178]. Overall, these features may contribute to favor bronchoconstriction and allergic responses in inflammatory lung diseases.

ATP-induced airway secretion has gained special attention in the last decade. The P2X4R is involved in mucous secretion and in the control of lung surfactant production [165,170]. Mechanical stretch of alveoli increases the release of ATP to the extracellular milieu along with increases in lung surfactant production [179,180]. Alveolar type II (AT II) cells are specialized in the secretion of lung surfactant [181]; these cells harbor the P2X4R as the most abundant P2X receptor subtype [182]. The P2X4R has a unique subcellular location in AT II cells, being found in lamellar bodies that contain lung surfactant. The release of lung surfactant is an exocytotic process designated by fusion-activated Ca2+ entry (FACE), which is operated by P2X4 (and possibly P2Y_2_) receptors activation [182]. The P2X4R inward current also drives water reabsorption across the alveolar epithelium, which is crucial to maintain alveoli relatively dry to increase gas exchange efficiency [183]. Although not completely resolved, it is believed that the main extracellular ATP source for P2X4R activation is alveolar type I (AT I) cells [179,180], yet extracellular release of ATP may be further amplified by P2X4R-induced lysosome fusion, resulting in the release of ATP stored inside lamellar bodies [184].

Acute lung inflammation causes a disproportionate increase in extracellular ATP, which contributes to dysregulation of surfactant production and initiation of the inflammatory response [185]. Although speculative, from a mechanistic point of view, alveolar P2X4Rs may have some role in acute respiratory distress syndrome (ARDS), a life-threatening condition frequently requiring emergent positive-pressure ventilation support, so it is pursued nowadays in the SARS-CoV-2/COVID-19 pandemic [186]. In this context, selective modulation of P2X4Rs activation may control lung surfactant production and protect patients from barotrauma and alveolar collapse, which are common features in ARDS [187,188]. Likewise, patients presenting with ADHF usually require non-invasive positive-pressure ventilation to resolve signs and symptoms of congestion. It is, thus, tempting to hypothesize that activation of alveolar P2X4Rs may also be beneficial to these patients’ outcomes.

## 7. Is there a Dark Side of P2X4 Receptor Activation Outside the Cardiovascular System?

In this section, we will briefly address putative side-effects of P2X4R activation outside the cardiovascular system, which might be relevant when translating the use of P2X4R agonists to the clinical practice.

The P2X4R was first identified in the central nervous system [21], with the following reports showing its widespread expression both in central and in peripheral neurons (reviewed in [9,189,190]. Great attention has been given to the modulation of P2X4R activity to control chronic pain. The P2X4R is upregulated in spinal microglia cells following peripheral nerve injury; genetic ablation or pharmacological blockage of overexpressed P2X4Rs can reverse allodynia occurring in this condition [191]. Increasing evidence demonstrates that blockade of the P2X4R alleviates neuropathic and inflammatory pain [45,192,193], as well as other causes of chronic pain, including migraine [194]. Hyperalgesia caused by P2X4R activation seems to depend on brain-derived neurotrophic factor (BDNF) release [192,195]. In this context, experimental data indicate that the P2X4R may play a role in long-term potentiation and synaptic plasticity [196,197], which may underlie its effect on chronic pain. Notwithstanding the involvement of the P2X4R in neuropathic and inflammatory pain conditions, its activation did not affect acute pain sensation in animals not prone to pain hypersensitivity [191]. Although changes in nociception may be a problem for the clinical use of P2X4R agonists in cardiovascular diseases, this warrants confirmation in clinical trials due to contradictory results using animal models. The same holds true regarding involvement of the P2X4R in neuroinflammatory conditions, as conflicting results, either protective [135,172] or deleterious [198,199], have emerged. With this in mind, forthcoming P2X4R agonists targeting the cardiovascular system should not cross the blood–brain barrier; otherwise, prolonged stimulation may have negative impacts in the course of some neurodegenerative diseases, chronic pain conditions, and epilepsy in susceptible individuals [8,9,199].

Rather than deleterious, the P2X4R expression and function in the brain reward system have been associated with salutary effects in alcohol abuse. Ethanol is a preferential inhibitor of the P2X4R, a situation that can be overcome by positive allosteric modulators, like ivermectin or derivates, which have been shown to reduce ethanol drinking behavior (reviewed in [200,201]).

Inflammatory flairs may also occur with P2X4Rs activation, particularly in patients with proinflammatory backgrounds, such as rheumatoid arthritis and asthma [8,9,166,173,202]. Pruritus and hypersensitivity reactions can also be a consequence of stimulation of P2X4R in mast cells and other immunocytes [42,166]. Cancer is another type of inflammatory condition where the P2X4R might be involved, although its role remains uncertain [8].

In the opposite direction of the inflammatory spectrum, activation of P2X4R in macrophages exhibited both anti-inflammatory properties and bactericidal activity against Gram-negative bacteria in the cecal ligation and puncture-induced sepsis rodent model. This P2X4R-mediated protection improved multi-organ failure and sepsis-related death [174], which supports future therapeutics with P2X4R agonists to treat patients with ADHF secondary to sepsis.

## 8. Conclusions

The development of novel purinergic-based medications has been struggling with the ubiquitous nature of P1 and P2 purinoceptors and lack of drug selectivity. Pushed by the success of P2Y_12_ receptor antagonists as anti-thrombotic drugs, last decades have provided new experimental and pharmacological tools to dissect the purinergic signaling cascade, including the purinoceptors function, which have granted a series of promising druggable targets, and some of them were already admitted to clinical trials. Selective drugs targeting the P2X4R are limited to few antagonists and positive allosteric modulators, without any selective agonist being available so far. Despite this, researchers worldwide have been unlocking the potential of the ionotropic P2X4R to treat several diseases. By reviewing recent data in the literature, we have highlighted here the benefits putatively arising from activation of the P2X4R, either by directly ameliorating the cardiac function or by indirectly improving the function of other systems impacting cardiovascular disease conditions. We also provide some hints about problems to be solved regarding unwanted side effects that may emerge upon activation of P2X4R to stimulate the cardiovascular system, such as arrhythmias and worsening of diastolic dysfunction in the heart; water–electrolyte imbalance and remodeling in renal injury; inflammation, mucous secretion, and hyperreactivity in airway diseases; and nociceptive-related changes. Taken together, we provide input for forthcoming development of medications based on selective activation of the P2X4R to reduce the burden of cardiovascular diseases.

## Figures and Tables

**Table 1:** Pharmacology of the P2X4 receptor.

	Compound	EC_50_ or IC_50_ (µM) *			P2X Cross-Reactivity/Fold Selectivity	References
		Human	Rat	Mouse		
**Agonist**	ATP	0.7–5.7	1.7–16.2	0.3–2.3		[12,17,21,22,25,26,27]
	ATPγS	10.9				[28]
	2-meSATP	0.3–2.2	~ 1–10	1.4		[21,27,28,29]
	CTP		~0.1–1 mM		P2X1,2,3,7	[21,30,31,32]
	BzATP	0.5–9.4	>100	2.9	P2X7 (h)	[12,27,28]
	α,β-meATP	0.8–19	≥100	7–>100	P2X1,3	[26,27,28]
	β,γ-meATP	>100	>100	>100		[27]
	AP4A^Ɣ^	0.1-3	20–>100	2.6–>100		[26,27,28]
	AR-C67085MX	2.5	>100	>100		[27]
	8-Azido-ATP	>100	>100	>100		[27]
	β,γ-Imido-ATP	6.5	>100	>100		[27]
	MRS-2339					[33]
	MRS-2978					[34]
	PSB-0412	2.1				[27]
**Antagonist**	PPADS	9.6–>100	>300	>100		[26,28]
	Suramin	>100	>100	>100		[26,28]
	KN-62	>10	>10	>10		[26]
	TNP-ATP	1.5	1.3–4.71	1.3–4.2	P2X1,2,3,7	[27,35,36,37,38]
	Brilliant Blue G	3–100			1000-fold more potent at P2X7 (r)	[39]
	5-BDBD	0.3–1.2	0.75–3.5	2.04	10-fold P2X1 (r), 3-fold P2X3 (r)	[27,36,40]
	BX-430	0.54–1			10 to 100-fold P2X1,3,5,7	[41,42]
	Carbamazepine der. ^†^	3.44	54.6	14.9	2 to 30-fold P2X1,2,3,7 (h)	[43]
	PSB-12054	0.19	2.10	1.8	≥50-fold P2X1,2,3,7 (h)	[35]
	PSB-12062	1.4	0.9	1.8	≥35-fold P2X1,2,3,7 (h)	[35]
	PSB-15417	10 ^‡^			>5-fold P2X7 (h)	[44]
	NP-1815-PX	0.26			P2X1,2,3,7	[45]
	NC-2600 ^Ʊ^					[46]
	UoS14919	61 nM				[47]
	Paroxetine	1.87–4.8	1.64–2.45	0.7		[27,48]
	Duloxetine	1.59–17				[49,50]
	BAY-1797	0.2				[51]
	IgG#151-LO ^||^	0.7			10,000-fold more potent over other P2X	[52]
**PAM**	Ivermectin	0.1			P2X7 (h)	[53,54]
	Cibacron blue	>300				[55]
	Ginsenosides	7.5–10.5			P2X7	[56]
	Testosterone butyrate		30 ^‡^		P2X2	[57]
	Zn^2+^		1.8			[58]
	Cd^2+^		7.3			[58]
	Alfaxalone		0.4–1.6			[59]
	Allopregnanolone		0.4			[59]
	Propofol	56				[39]
**NM**	Ethanol		5–200 mM ^‡^		PAM at rat P2X3	[60,61]
	H ^+^	pKa ~6.8			inhibits P2X1,3,4; stimulates P2X2	[62]
	Hg^2+^		9			[58]
	Cu^2+^		8.6			[58]
	Fluvastatin ^¥^	10 for 1 h				[63]
	Filipin III ^¥^	10 for 30 min				[63]
	Methyl-β-cyclodextrin ^¥^	10 mM, 1 h				[63]
	t-DCA ^§^	160				[64]

Data are mostly from functional studies (e.g., electrophysiological currents, intracellular calcium oscillations) in heterologous systems expressing human (h), rat (r) or mouse (m) P2X4 receptors. * Potency of agonists and positive allosteric modulators (PAM) is represented as half maximal effective concentration (EC_50_), whereas for antagonists and negative modulators (NM) indicated is the half maximal inhibitory concentration (IC_50_). EC_50_ and IC_50_ values are in µM, unless stated otherwise. ^Ɣ^ AP4A, adenosine tetraphosphate. ^†^ N,N-diisopropyl-5H-dibenzo[b,f]azepine-5-carboxamide. ^Ʊ^ NC-2600 is a specific P2X4 antagonist from *Chemifar^®^*, with unknown structure and selectivity, currently in phase I clinical trial for neuropathic pain. ^‡^ Potency not calculated, it indicates the concentration of the compound used to produce the functional effect. ^¥^ Activity suppressed by cholesterol depletion. ^§^ Tauro-deoxycholic acid (t-DCA). ^||^ Mouse antibody anti-P2X4 IgG#151-LO.
